# Editorial Correspondence

**Published:** 1879-12

**Authors:** J. C. LeHardy

**Affiliations:** Savannah


### EDITORIAL CORRESPONDENCE.

*Dr. J. G. Westmoreland*—Dear Sir: In fulfilment of my promise I give you a bird's-eye view of the American Public Health Association, as it appeared to me at its recent session in Nashville; and also some reflections and suggestions of my own, which I hope you may consider pertinent.

This meeting was a grand success. There were two hundred members paying into the treasury $1,000. To Dr. J. Berrien Lindsley, of Nashville, the energetic and genial local secretary, the association is mostly indebted for this success.

As you are aware, the A. P. H. A., and the National Board of Health met at the same time and place for purposes closely allied. The working element of the Executive Committee of these two bodies was the same, and very efficient. Doctor Billings, of the U. S. A., assisted by Dr. Turner, of the Navy, detailed for special service, have certainly devoted their energies to the work, and they have done full justice to themselves and to the supporters of Central Quarantine.

The election of Dr. Billings as President of the A. P. H. A., was therefore a well deserved honor. The first three days of the meeting were spent in the reading of various papers approved by the Executive Committee, and in a prolific discussion of these on the fourth and last day, when a large number of the members had left for their homes, the real, or rather the practical business was transacted, the members voting on that day being at times less than fifty.

The inspectors of quarantine, etc., appointed by the National Board of Health, were fully represented, and very interesting descriptions of the work done this year were given by several of these gentlemen. They failed, however, to give us the full list and the names of the many localities where yellow fever made its appearance in spite of the strict quarantine enforced at Memphis and along the Mississippi river by the national as well as the local authorities. Only one paper was read in support of the indigenous origin of yellow fever, and its author was at the time in California. Had he been in person at the meeting he would very likely have followed the good example of others in saying not a word. The quarantinists have gained a great victory. They have gone home with the pleasant feeling that they are masters of the field, and that by the passage of the resolutions framed by the Executive Committee—*before mentioned*—they have given to the National Board of Health all the power which could be conferred upon them by the moral support of so respectable a body as the American Public Health Association. This I consider a happy termination of a well regulated meeting; for it may become the means of settling in two or three years the vexed question of exotic or indigenous origin of yellow fever. But to attain this very desirable result the National Board of Health must necessarily do their full duty.

They must carry out well digested quarantine regulations in such manner that "no low, long, black hulled schooner shall arrive from the West Indies (or elsewhere) to afford an easy solution."—(Arnold). They must so quarantine vessels, people and things (by their own devised methods) at all our ports and inlets, that not one *germ* (or what not) of the disease shall enter, *alive*, this benighted land.

Boston, New York, Philadelphia, Baltimore, Norfolk, must be guarded strictly, as well as the more Southern ports, because the germ can be and is imported in those localities by the same vehicles as it is here.

With such a quarantine we will, according to the views expressed by importationists, be completely and entirely safe from further invasions of yellow fever epidemics, for they now say that the yellow fever germ may live and propagate in this country for *one year*, as it did in Memphis and elsewhere during the past summer and fall, and that if, after the expiration of one year, the disease should make its appearance again, it must have originated from freshly imported germs. Then the last germ must have disappeared from the United States, and the National Board must forever keep out the foreign intruder by enforcing their quarantine regulations with rigor and impartiality.

But I would nevertheless advise, as the quarantinists themselves are very prone to do, that the people of the yellow fever zone be ever on the watch, not only keeping their own yards, privies and wells clean and pure, and their lands well drained, but get their neighbors do the same, and see to it that their State and municipal authorities perform the duty of public sanitation in a prompt and thorough manner. Let the authorities of every city, town and village—let every doctor faithfully and promptly report each and every case of yellow fever which may occur; let them investigate thoroughly into the origin and the causes for themselves; call upon the nearest *national inspector* to be present and to take part in the investigation, and publish at once through the public press the result of their investigations. Let all of this be done and the people will be safe, whether yellow fever is exotic or indigenous. On the other hand, if sanitation be neglected it will be, (though strict quarantine be enforced) at the expense of great sufferings and pecuniary loss to this country.

My views are, Doctor, that quarantine and the inspections incident thereto, have acquired an importance which they do not deserve. National bodies like the A. P. H. A., and the National Board of Health, should take cognizance of every branch of sanitation, and in their estimation measures for prevention should rank in accordance with the amount of good they may be expected to accomplish in preserving the public health and preventing mortality. *They should have no concern for commercial or political interests*, but should devote themselves strictly to efforts for the prevention of avoidable diseases; and to this end they should carefully ascertain the number of deaths which have been caused in the last one hundred years by the various preventable diseases, and what per centage of the whole mortality was due to each of these diseases. A table prepared on this plan would show results something like this:

Mortality by yellow fever and cholera......... 120,000

Malarial fevers.............................. 10,000,000
Typhoid fever and other filth diseases......... 10,000,000

From data thus obtained, a table should be prepared in which each disease, and each cause of disease, should rank according to its influence upon the vital energies of the population and the amount of mortality due directly, or indiretly, to it. By this and other means, the work of sanitation would be brought into shape and laid out before them. As the amount of mortality from yellow fever is but a small fraction of that produced by malarial fever and filth diseases, yellow fever should not out-rank our great destroyer of life. Malarial fever should stand foremost on the list, and ever bring before the National health authorities, the fact, that in a sanitary point of view, drainage is in importance far above quarantine. And to these points, I would here call the attention of our legislators, in order that they may so amend the laws under which the members of the National Board of Health are acting, as to enable them to direct their efforts and to expend the money intrusted in ther keeping by Congress, in such sanitary works as will reduce the rate of mortality in those places as show the greatest need for its diminution.

Let them furnish sanitary surveys and estimates to the authorities of towns and villages decimated by malarial or filth diseases, or subject to epidemics of infectious diseases; let them guide the work of sanitation by their counsels, and wherever the money necessary to complete the work is wanting, let them disburse of their fund in proportion to the amount already expended by the local authorities, to the population or the benefits to be derived from such work. This will secure for the National Board the co-operation of State and local Boards of Health, and the good will of the people.

Very truly yours,

J. C. LeHARDY.

SAVANNAH, DEC., 2, 1879.

